# Stress and vision-related quality of life in acute and chronic central serous chorioretinopathy

**DOI:** 10.1186/s12886-020-01361-9

**Published:** 2020-03-06

**Authors:** Meenakshi Kumar, Elon H. C. van Dijk, Rajiv Raman, Pooja Mehta, Camiel J. F. Boon, Abhilash Goud, Seelam Bharani, Jay Chhablani

**Affiliations:** 1 Shri Bhagwan Mahavir Vitreoretinal Services, 18 College Road, Sankara Nethralaya, Chennai, Tamil Nadu 600 006 India; 2 Department of Ophthalmology, Leiden University Medical Center, Leiden, The Netherlands; 3 Srimati Kannuri Santhamma Centre for Vitreo-Retinal Diseases, L. V. Prasad Eye Institute, Hyderabad, Telangana India; 4 Department of Ophthalmology, Amsterdam UMC, University of Amsterdam, Amsterdam, Netherlands

**Keywords:** Central serous chorioretinopathy, Cohen’s perceived stress scale, Stress, Visual function questionnaire, Quality of life

## Abstract

**Background:**

To compare vision-related quality of life (VRQOL) between acute and chronic Central serous chorioretinopathy (CSC) and correlate this with Cohen’s Perceived Stress Scale (PSS) questionnaire.

**Methods:**

Patients who were diagnosed with both acute and chronic CSC were recruited in this study. Vision-related quality of life (VRQOL) was assessed with Rasch revised National Eye Institute Visual Functioning Questionnaire 25 (NEI-VFQ25) and perceived stress with Cohen’s PSS questionnaire in 118 subjects with either acute or chronic CSC. The quality of life score was compared between patients with acute and chronic CSC. Correlations between the functional score and visual acuity (VA), stage of CSC, and stress were studied.

**Results:**

There was no significant difference in VRQOL between Acute and Chronic CSC. In Acute CSC, affected eye VA correlated significantly with near vision question of the visual function subscale. Better eye VA correlated significantly with distance vision, social function, role limitation and dependency of the socioeconomic subscale. In chronic CSC, affected eye VA correlated with social function question of the socioemotional subscale and the better eye VA correlated with driving and distance vision of the visual function subscale. No other significant correlations with VA were noted. No correlations were observed between outcome of Cohen’s PSS questionnaire and NEI-VFQ25 scores of acute and chronic CSC.

**Conclusion:**

The **V**RQOL is similar between acute and chronic CSC. Perceived stress was not found to influence the VRQOL in CSC.

## Background

Central serous chorioretinopathy (CSC) is a self-limiting disease with spontaneous resolution of serous retinal detachment (SRD) in a noteworthy percentage of patients, and in these cases usually associated with recovery of good vision [1]. However, in recurrent and chronic cases of CSC, vision may be compromised due to damage to the photoreceptors [1, 2] .Vision-related quality of life (VRQOL) can be assessed using the National Eye Institute Visual Function Questionnaire-25 (NEI-VFQ25). The reliability and validity of the questionnaire has been established among patients with various other conditions such as age-related macular degeneration, [3, 4] retinal venous occlusions, [5] uveitis, [6] retinitis pigmentosa [7], and diabetic retinopathy [8]. Decreased VRQOL has been reported both in acute and chronic CSC [9, 10].

However, comparison of VRQOL for acute versus chronic CSC has not been previously reported. Although, the association between psychological stress in CSC and VRQOL has investigated [11] multiple occasions, the quality of life and the perceived stress in these patients has not been explored. The aim of the present study is to compare the VRQOL in acute Vs Chronic CSC and to correlate these scores to perceived stress as determined by Cohen’s perceived stress scale (PSS).

## Methods

Between January 2016 and December 2016, 118 patients with a diagnosis of CSC were recruited in this study. Written informed consent was obtained from the subjects and the study was approved by organisation’s Institutional Review Board (Ethics Committee: L V Prasad Eye Institute Ethics Committee) and was performed according to the tenets of Declaration of Helsinki. The diagnosis of CSC was established by the retinal specialist based on comprehensive ophthalmic examination including best-corrected visual acuity (VA), slit lamp examination, fundus examination, and features based on optical coherence tomography (OCT), fluorescein angiography (FA), and if deemed necessary indocyanine green angiography (ICGA). Acute CSC was defined as SRD through a focal pinpoint leak in the retinal pigment epithelium, which typically resolves within 3–4 months from the onset of symptoms. Chronic CSC was characterised by widespread Retinal Pigment Epithelium (RPE) abnormalities with or without SRD, associated with several active leakage sites, and an onset of symptoms more than 3–4 months ago [2, 12, 13]. Patients with a history of any other ocular disease were excluded from the study.

Before treatment, all patients completed the NEI-VFQ25 and Cohen’s PSS.

### Measurements of VRQOL and perceived stress

The Rasch corrected NEI-VFQ25 was used to assess VRQOL in our study participants. The NEI-VFQ25 was applied to all patients face-to-face in a quiet room in the hospital. The original form of NEI VFQ questionnaire has 25 questions with 12 subscales: general health, general vision, ocular pain, near activities, distance activities, vision-specific social functioning, vision-specific mental health, vision- specific role difficulties, vision-specific dependency, driving, colour vision, and peripheral vision. The patients were asked to rate the level of severity of visual symptoms or difficulty of activities on a 5 point rating in the questionnaire [14]. The long form Rasch corrected version of the questionnaire described by Pesudov K et al [15] was used in this study and it has 18 questions categorized into two over all subscales, measuring visual function scale and one socioemotional scale. The 5-point rating in the questionnaire was then entered in a ready to use Rasch converted excel described by the author as raw scores, which gives Rasch corrected scores for the questions in which higher negative logit indicated better VRQOL. The two subscales had individual mean scores which are average scores from each subscale. The PSS is a standardised self-report questionnaire of globally perceived stress. Six of the questions are negative (e.g., “How often have you felt nervous or stressed?”), and the remaining 4 are positive (e.g., “How often have you felt that things were going your way?”). Each item has to be rated for the past month on a 5-point Likert-type scale (1 = never to 5 = very often). In scoring the measure, the 4 positive items are reversed scored, and then all the items are summed. A higher total score indicates greater stress [16]. Both surveys were done before treatment.

### Statistical analyses

Statistical analyses were done using SPSS (IBM SPSS 22, IBM Corp., Armonk, USA). The normality assumption was checked using the Shapiro-Wilks test. For comparison of means between acute CSC and chronic CSC, Mann-Whitney U test was used. For correlation between VA, stress scale, and composite score of VRQOL, Spearman’s correlation was used. Categorical data such as gender distribution was analysed using Chi-square test. For statistical analysis, Snellen VA was converted to Logarithmic minimum angle of resolution (LogMAR)VA. The statistically significant level was *p* < 0.05.

## Results

Of the 118 patients recruited for the study, 53 had acute CSC and 65 had chronic CSC. The age, duration of disease symptoms before diagnosis, the VA in the worse and better eye are shown in Table 1.

**Table 1:** Characteristics of study subjects

	Acute CSC	Chronic CSC	***p*** value
Age (years)	39.5 ± 9.0	45.6 ± 9.2	**0.000**
Duration of disease (months)	1.27 ± 1.06	37.30 ± 52.24	**0.000**
Gender (M/F)	47/4	59/6	0.246
BCVA^a^ worse eye (logMAR)	6/6–6/24(0.00–0.60)	6/6–6/18 (0.00–0.477)	0.460
BCVA^a^ better eye (logMAR)	6/5–6/9(− 0.07–0.176)	6/5–6/9(− 0.07–0.176)	0.567

^a^*BCVA* best-corrected visual acuity (Values within bracket indicate logMAR vision)

The mean age of the patients with acute CSC was 39 years (range 31–48 years) and chronic CSC was found to be 45 years (range 36–54). The mean age difference between the groups was found to be significantly different and higher in the chronic CSC group (*p* = 0.000). Although different worst eye vision was observed between the groups, the VA was not significantly different between the groups.

The Rasch corrected NEI-VFQ25 scores from each question was compared between the acute and chronic CSC (Table 2). There was no significant difference between the VRQOL score of Acute and Chronic CSC.

**Table 2:** Comparison between outcome of the NEI-VFQ25 for acute and chronic CSC patients with respect to reference group

Measurement	Questions used to measure	Acute	Chronic	Acute	Chronic	^**a**^***∆***between acute and chronic CSC	***p*** value
		Median	Median	Mean (SD)	Mean (SD)		
Visual functional scale	Q2 General Vision	1.23	1.23	2.0993 (1.823)	1.2847 (3.205)	0.8146	0.314
	Q5 Near Vision	−0.9	−2.98	−2.1949 (2.372)	− 2.8196 (2.004)	0.6247	0.374
	Q6 Near Vision	−4.91	− 4.91	−3.644 (2.130)	−4.147 (1.258)	0.503	0.576
	Q7 Near Vision	−3.7	− 3.7	− 3.123 (1.247)	− 3.3542 (0.805)	0.2312	0.991
	Q8 Distance Vision	−4.28	− 4.28	−3.264 (2.010)	− 3.3933 (1.500)	0.1293	0.379
	Q9 Distance Vision	−4.84	−4.8	− 4.1609 (1.596)	− 4.3049 (1.315)	0.144	0.613
	Q14 Distance Vision	−3.41	− 3.41	−2.074 (3.165)	− 3.0195 (1.692)	0.9455	0.57
	Q15 Driving	−3.57	−3.57	− 3.2793 (0.8774)	−3.2247 (0.90043)	− 0.0546	0.287
	**Mean score**	−2.937	− 2.937	− 2.44 (1.200)	− 2.680 (1.118)	0.2396	0.538
Socioemotional scale	Q11 Social Function	−2.76	−2.76	− 2.541 (0.626)	−2.556 (0.697)	0.015	0.645
	Q 13 Social Function	−2.39	− 2.39	− 2.1796 (0.825)	−2.2172 (0.562)	0.0376	0.168
	Q 17 Role Limitation	−5.1	−5.1	−4.7889 (1.040)	− 4.68 (1.396)	−0.1089	0.459
	Q18 Role Limitation	−4.63	−4.63	− 4.3191 (1.201)	−4.1395 (1.568)	−0.1796	0.337
	Q20 Dependency	− 2	− 2	−1.7527 (1.045)	− 1.8496 (0.6722)	0.0969	0.611
	Q21 Mental Health	−4.01	− 4.01	−3.896 (0.567)	− 3.92 (0.505)	0.024	0.81
	Q22 Mental Health	−3.68	−3.68	− 3.5687 (0.421)	−3.4553 (0.952)	−0.1134	1
	Q23 Dependency	−2.5	−2.5	− 2.4231 (0.515)	− 2.4414 (0.719)	0.0183	0.807
	Q24 Dependency	−2.29	−2.29	− 2.1367(.719)	−2.1689 (0.640)	0.0322	0.933
	Q25 Mental Health	−2.32	−2.32	− 2.2431 (0.515)	−2.2593 (0.458)	0.0162	0.528
	**Mean score**	−3.16	−3.168	−2.840 (0.757)	−2.758 (0.871)	−0.082	0.869

^a^∆: Mean Difference

The correlation between subscales, mean scores in NEI-VFQ25 and vision in both the better and worse eye in acute and chronic CSC was performed (Table 3). In acute CSC, one question on near vision showed a positive correlation with affected eye vision. The distance vision showed positive correlation with better eye vision. In chronic CSC, driving and distance vision question showed a significant correlation with vision in the better eye. The social function question showed significant positive correlation with better eye vision in acute CSC and with affected eye in chronic CSC. In acute CSC, a positive correlation was noted on role limitation questions, dependency and the mean score and fellow eye vision. No other statistically significant correlations were observed, neither for acute CSC nor for chronic CSC.

**Table 3:** Spearman’s Correlation between the subscales and the composite score for the NEI-VFQ25 with vision for both eyes in acute and chronic CSC

NEI VFQ 25^**a**^	Correlations (Spearman)	Acute CSC				Chronic CSC			
		Affected eye		Fellow eye		Affected eye		Fellow eye	
		Rho ^b^	p	Rho ^b^	p	Rho ^b^	p	Rho ^b^	p
Visual functions score	Q2 General vision	−0.062	0.663	0.109	0.439	0.166	0.218	−0.216	0.581
	Q5 Near Vision	0.153	0.283	0.071	0.616	0.036	0.789	−0.096	0.446
	Q6 Near Vision	−0.195	0.169	0.156	0.271	0.092	0.499	0.108	0.397
	Q7 Near Vision	**0.339**	**0.014**	0.174	0.214	0.171	0.208	0.198	0.116
	Q8 Distance Vision	0.021	0.883	0.111	0.429	0.184	0.173	0.074	0.564
	Q9 Distance Vision	−0.088	0.545	0.061	0.669	0.146	0.284	0.136	0.283
	Q14 Distance Vision	0.057	0.689	**0.551**	**0.000019**	−0.032	0.814	**0.371**	**0.003**
	Q15 Driving	0.021	0.884	0.166	0.238	0.085	0.529	**0.313**	**0.011**
	**Mean score**	−0.039	0.782	0.196	0.16	0.147	0.275	0.013	0.919
Socioemotional score	Q11 Social Function	0.097	0.494	**0.281**	**0.042**	**0.291**	**0.03**	0.199	0.115
	Q13 Social Function	0.196	0.168	0.116	0.412	0.092	0.5	0.162	0.2
	Q17 Role Limitation	−0.205	0.144	**0.332**	**0.015**	0.086	0.527	0.05	0.694
	Q18 Role Limitation	−0.179	0.204	**0.379**	**0.005**	−0.001	0.993	0.058	0.648
	Q20 Dependency	−0.018	0.901	**0.307**	**0.027**	−0.148	0.275	0.192	0.129
	Q21 Mental Health	0.057	0.689	−0.128	0.365	−0.1	0.471	0.134	0.304
	Q22 Mental Health	0.044	0.758	0.05	0.727	−0.044	0.747	−0.051	0.696
	Q23 Dependency	−0.24	0.9	**0.413**	**0.002**	−0.188	0.178	−0.106	0.416
	Q24 Dependency	−0.08	0.575	**0.3**	**0.029**	−0.224	0.104	−0.008	0.952
	Q25 Mental Health	0.176	0.218	0.143	0.311	0.238	0.086	−0.086	0.511
	**Mean score**	−0.003	0.981	**0.296**	**0.031**	0.115	0.394	0.095	0.45

^a^*NEI-VFQ25* National Eye Institute Visual Functioning Questionnaire-25, ^b^Rho: Spearman’s correlation

Correlation was performed between the mean scores and the stress scales. In the both acute and chronic CSC stage, the stress and both Visual function mean score and Socialemotional mean score did not correlate significantly (Acute Visual function r = 0.182, *p* = 0.193, socioemotional r = 0.091, *p* value: 0.518, Chronic visual function r = − 0.066,*p* = 0.601, socioemotional score r = − 0.051, *p* = 0.688)) first visit. Chi-square test was performed by categorising the Cohen’s PSS into less stressed (≤ 20) and more stressed (> 20) and acute and chronic cases. There was no statistical significance between stage of CSC and the stress score (*χ*^2^ = 0.009, *P* = 0.925).

## Discussion

Based on the results of the current study, we conclude that VRQOL is not significantly different between acute and chronic CSC.

In this current study, the mean scores are seen in negative logits. Previous literature indicates, the more negative the logit score, the better is the score [17]. Assessing the scores in both acute and chronic CSC, the VRQOL is not much affected in CSC upon comparison to other debilitating macular disease. Although previous studies have used the conventional NEI VFQ25 scoring, we are reporting the scores of other macular diseases for overall comparison. The composite score in other maculopathies such as diabetic retinopathy (52.8 ± 19.0), diabetic macular edema (53.0 ± 20.5), branch retinal vein occlusion (54.7 ± 15.5), central retinal vein occlusion (60.4 ± 17.6) macular hole (71.2 ± 14.3), and epiretinal membrane (66.9 ± 10.5) has been described to show lower scores than reported data in literature for CSC [5] [9, 10]. Moreover, the reduction of the VRQOL in CSC has been found to be less pronounced in comparison with other maculopathies[65.3 ± 8.7][73.3 ± 10.44], which is in line with our study [10, 13, 2]. Additionally, it could also be speculated that NEI VFQ’s questions are not specific to the impact of CSC on eyes.

In CSC, both in the acute and chronic type, the lowest scores (positive logit) were observed in general vision question. Symptoms such as relative central scotomas, reduced vision, metamorphopsia, and distortion can severely disturb image quality. Patients who have been included in this study predominantly had unilateral CSC, which could cause only a slight reduction in VRQOL. Moreover, the visual expectancy could be relatively high in younger patients, which could cause the discrepancy between perception and actual visual function [8]. However, in general, the presence of central serous chorioretinopathy doesn’t seem to affect the quality of life much in both acute and chronic state of the disease.

The comparison of NEI-VFQ25 score to VA yielded few significant correlations in the visual function score in both acute and chronic CSC. In the acute state, the affected eye showed correlation with near vision and distance vision questions which signifies some activities like searching for things in a crowded shelf and going out to see events (Near and distance vision based tasks) are affected if the affected eye vision is reduced drastically. If the vision is reduced marginally then the fellow eye is enough to manage daily activities [18].

However, in acute CSC, many questions corresponding to the socioemotional function showed significant correlations with the fellow eye vision such as Social functioning, Role limitation and Dependency indicating if they have good vision in the fellow eye, the socioemotional elements are better. This agrees with previous ocular quality of life studies where similar findings were observed [19].

In Chronic CSC, driving and distance vision were the only scales correlating with fellow eye vision indicating even if the affected eye vision is bad, if they have good vision in the fellow eye, they will be able to adapt. This is true in case the vision reduction is mild in the fellow eye, due to binocular summation [20]. In general, socioemotional score were less affected in the chronic CSC than Acute CSC where only one element, social functioning, was found to correlate with affected eye vision.

Even though VA was reduced in our patients, it did not correlate with multiple elements in the visual functions scores, which is in line with previous studies and supports the notion that visual impairment does not solely contribute to VRQOL [21, 22]. In most acute CSC patients, the vision can be optimized with refraction, but the quality of vision apparently remains affected due to distortion and metamorphopsia [23].

The Cohen’s PSS score was correlated with the mean scores in acute and chronic CSC. There are many known risk factors for CSC, such as corticosteroid use, systemic lupus erythematosus, and hypertension [24]. Under all these conditions, stress hormone is released. Acute CSC patients have been shown to both have elevated cortisol levels [25], while some studies show no correlation [26]. In chronic CSC, Tufan et al. found normal cortisol levels [27]. Although this is not a direct measurement of stress, in the current study questionnaire-based assessment of stress was performed, where no significant correlation was observed between mean score in acute and chronic CSC and Cohen’s PSS. The Cohen’s PSS only expresses the qualitative aspect of stress and is not a direct measure of cortisol. Thus, the result can get altered with the perspective of the patient. A study by van Haalen et al., agrees with our study finding suggesting that chronic CSC patient have many adaptive strategies and coping mechanisms to overcome stress and it is not necessarily the causative [28].

Our study has certain limitations. Due to the absence of control group data, the VRQOL was not compared any normal reference. The comparison of the data with an age-matched control group would generate a better idea about the VRQOL of patients with CSC. However, this is the first study to directly compare VRQOL between acute and chronic CSC. A follow-up study with larger sample size would be of importance to validate our findings.

## Conclusion

In conclusion, we found no difference in VRQOL between acute and chronic CSC patients in the current study. We also found that the perceived stress was not significantly difference for both acute and chronic CSC and that perceived stress didn’t influence the VRQOL.

## Data Availability

The datasets generated and/or analysed during the current study are not publicly available as it is against the institutional policy but are available from the corresponding author on reasonable request.

## Abbrevations

CSC: Central serous chorioretinopathy
FA: Fluorescein angiogram
ICGA: Indocyanine green angiography
LogMAR: Logarithmic minimum angle of resolution
NEI VFQ25: National Eye Institute Visual Functioning Questionnaire 25
OCT: Optical coherence tomography
PSS: Perceived Stress Scale
RPE: Retinal Pigment Epithelium
SRD: Serous Retinal Detachment
VA: Visual Acuity
VRQOL: Vision related Quality of life
